# Designing, analyzing, and interpreting observational studies of physical activity and cancer outcomes from a clinical oncology perspective

**DOI:** 10.3389/fonc.2023.1098278

**Published:** 2023-04-14

**Authors:** Kerry S. Courneya, Christine M. Friedenreich

**Affiliations:** ^1^ Faculty of Kinesiology, Sport, and Recreation, College of Health Sciences, University of Alberta, Edmonton, AB, Canada; ^2^ Department of Cancer Epidemiology and Prevention Research, Alberta Health Services, Calgary, AB, Canada; ^3^ Departments of Oncology and Community Health Sciences, Cumming School of Medicine, University of Calgary, Calgary, AB, Canada

**Keywords:** cancer treatment, epidemiology, exercise, observational studies, physical activity, research methods, survival

## Abstract

Observational studies may play an important role in evaluating physical activity (PA) as a cancer treatment; however, few studies have been designed, analyzed, or interpreted from a clinical oncology perspective. The purpose of the present paper is to apply the Exercise as Cancer Treatment (EXACT) Framework to assess current observational studies of PA and cancer outcomes from a clinical oncology perspective and provide recommendations to improve their clinical utility. Recent systematic reviews and meta-analyses of over 130 observational studies have concluded that higher prediagnosis and postdiagnosis PA are associated with lower risks of cancer-specific and all-cause mortality. Most of these studies, however, have: (a) included cancer patients receiving heterogeneous treatment protocols, (b) provided minimal details about those cancer treatments, (c) assessed PA prediagnosis and/or postdiagnosis without reference to those cancer treatments, (d) reported mainly mortality outcomes, and (e) examined subgroups based on demographic and disease variables but not cancer treatments. As a result, current observational studies on PA and cancer outcomes have played a modest role in informing clinical exercise trials and clinical oncology practice. To improve their clinical utility, we recommend that future observational studies of PA and cancer outcomes: (a) recruit cancer patients receiving the same or similar first-line treatment protocols, (b) collect detailed data on all planned and unplanned cancer treatments beyond whether or not cancer treatments were received, (c) assess PA in relation to cancer treatments (i.e., before, during, between, after) rather than in relation to the cancer diagnosis (i.e., various time periods before and after diagnosis), (d) collect data on cancer-specific outcomes (e.g., disease response, progression, recurrence) in addition to mortality, (e) conduct subgroup analyses based on cancer treatments received in addition to demographic and disease variables, and (f) interpret mechanisms for any associations between PA and cancer-specific outcomes based on the clinical oncology scenario that is recapitulated rather than referencing generic mechanisms or discordant preclinical models. In conclusion, observational studies are well-suited to contribute important knowledge regarding the role of PA as a cancer treatment; however, modifications to study design and analysis are necessary if they are to inform clinical research and practice.

## Introduction

Physical activity (PA) may play an important role as a cancer treatment; however, few studies have been designed from a clinical oncology perspective (1). It is critical for exercise oncology researchers to recapitulate the key features of a clinical oncology setting if exercise is to be implemented as a cancer treatment in clinical practice (2). Importantly, this exhortation applies equally to preclinical, observational, and clinical studies (1). In general, preclinical animal studies in exercise oncology have recognized the importance of recapitulating clinical oncology scenarios (2) even if some of the clinical scenarios may be technically challenging. Moreover, a limited number of clinical trials have examined exercise as a cancer treatment within specific clinical oncology scenarios (3), however, sample sizes are generally inadequate and larger trials may be logistically challenging (4). Consequently, observational studies may be best positioned to generate clinical knowledge on PA as a cancer treatment. Unfortunately, these studies have been the least likely to be designed, analyzed, or interpreted from a clinical oncology perspective (1). As a result, current observational studies on PA and cancer outcomes have informed general guidelines for cancer prevention and survivorship (5–7) but have played a more limited role in informing clinical exercise trials (8) and clinical oncology practice (9). The primary purpose of the present paper is to evaluate current observational studies of PA and cancer outcomes from a clinical oncology perspective using the Exercise as Cancer Treatment (EXACT) Framework (1) and to provide recommendations for improving their clinical utility.

## The exercise as cancer treatment framework

**Figure 1:**
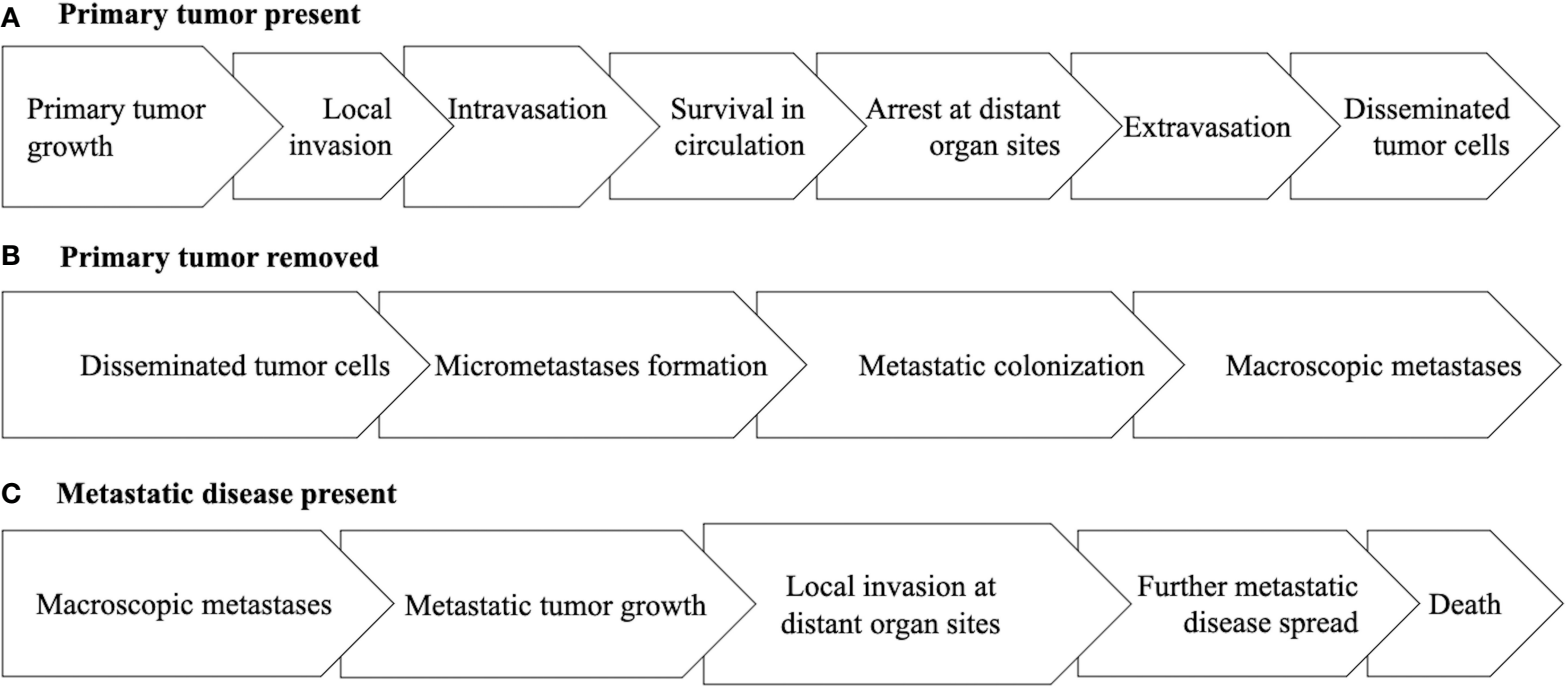
Diagram of the main steps of the metastatic cascade under the clinical oncology scenarios of the primary tumor being present **(A)**, the primary tumor being removed **(B)**, or metastatic disease being present **(C)**.

The EXACT Framework was proposed to organize and characterize the critical aspects of a clinical oncology setting to allow for a more systematic approach to the study of exercise as a cancer treatment across a wide range of cancers and treatment protocols (1). The EXACT Framework proposes nine generic clinical oncology scenarios based on two key clinical oncology variables at the time of the proposed exercise treatment—tumor/disease status and treatment status. For tumor/disease status, the clinically relevant issue is whether the primary tumor has been surgically removed, not surgically removed, or whether metastatic disease is present (**Figure 1**). Tumor/disease status is important because it highlights the steps along the metastatic cascade that exercise must affect to have a clinical benefit (10). Moreover, it acknowledges the genetic and epigenetic differences between primary tumors and metastatic disease (11, 12).

If the primary tumor is present, the main goal of exercise is to treat the early steps of the metastatic cascade focused on how the primary tumor progresses to disseminated tumor cells (DTCs) including primary tumor growth, local invasion, intravasation, survival of circulating tumor cells (CTCs), arrest of CTCs at distant organ sites, and extravasation (10). If the primary tumor has been surgically removed, the main goal of exercise is to treat the latter steps of the metastatic cascade focused on how DTCs progress to macroscopic metastases including micrometastasis formation and metastatic colonization (10). If macroscopic metastatic disease is present (including hematologic cancers), the main goal of exercise is to treat the final steps of the metastatic cascade focused on how limited macroscopic metastatic disease progresses to cause death including continued metastatic tumor growth, spread to other vital organs, and invasion at distant organ sites (13).

For treatment status, the clinically relevant issue is whether the extant disease (primary tumor, “micrometastases”, and/or metastases) has not been treated yet (treatment naïve), is currently being treated (active treatment), or has already been treated (previously treated). It is also possible that actively treated disease has been previously treated (i.e., second-line or later therapies) and that previously treated disease has been treated multiple times (i.e., heavily pretreated disease). Treatment status is important because existing and previous cancer treatments may alter the biology, genetics (e.g., newly acquired mechanisms of resistance), and/or location of any remaining cancer (14, 15) and modify the effects of exercise. Conversely, exercise may alter the biology and genetics of cancer and modify the effects of subsequent cancer treatments.

Exercise before a cancer treatment tests the direct effects of exercise on treatment naïve disease and establishes whether exercise alters the subsequent effects of a cancer treatment. Exercise during a cancer treatment tests the direct effects of exercise on actively treated disease and establishes whether exercise alters the effects of a concurrent cancer treatment. Exercise after a cancer treatment tests the direct effects of exercise on previously treated disease and establishes whether previous cancer treatments alter the subsequent effects of exercise. **Table 1** describes how different study designs may test the effects of treatment sequencing in relation to exercise.

**Table 1:** Proposed methodology for addressing treatment sequencing effects of exercise across different study designs.

Study design	Exercise before a cancer treatment	Exercise during a cancer treatment	Exercise after a cancer treatment
Preclinical (*in vitro*)	Cancer cells exposed to exercise or control serum prior to being exposed to a cancer treatment in a static (e.g., well plates) or dynamic (e.g., microfluidic system) environment	Cancer cells exposed to exercise or control serum at the same time as being exposed to a cancer treatment in a static (e.g., well plates) or dynamic (e.g., microfluidic system) environment	Cancer cells exposed to exercise or control serum after being exposed to a cancer treatment in a static (e.g., well plates) or dynamic (e.g., microfluidic system) environment
Preclinical (animal)	Animals with cancer randomized to exercise or no exercise prior to receiving a cancer treatment	Animals with cancer randomized to exercise or no exercise during a cancer treatment	Animals with cancer randomized to exercise or no exercise after receiving a cancer treatment
Observational (human)	Patients with cancer assessed for exercise levels (objectively or using self-report) prior to receiving a cancer treatment	Patients with cancer assessed for exercise levels (objectively or using self-report) during a cancer treatment	Patients with cancer assessed for exercise levels (objectively or using self-report) after receiving a cancer treatment
Clinical (human)	Patients with cancer randomized to exercise or no exercise prior to receiving a cancer treatment	Patients with cancer randomized to exercise or no exercise during a cancer treatment	Patients with cancer randomized to exercise or no exercise after receiving a cancer treatment

Although not previously discussed in the EXACT Framework, treatment status also applies to exercise. That is, at the time of the proposed exercise treatment, the tumor/disease may also be exercise naïve, currently treated with exercise, or previously treated with exercise. Similar to biomedical cancer treatments, exercise treatment status may have an effect on the efficacy of a proposed exercise treatment and/or subsequent biomedical treatments. Tumor/disease that has been previously or currently treated with exercise may be different than exercise naïve tumor/disease and may not be sensitive to further exercise treatment (even if exercise initially slowed the tumor growth and spread). Moreover, previous treatment of tumor/disease with exercise may make future biomedical cancer treatments more or less effective. Treatment sequencing is a critical issue in clinical oncology (16, 17) and must be addressed when integrating exercise into existing cancer treatments (**Table 2**).

**Table 2:** Exercise treatment status as part of the cancer treatment sequence.

Cancer treatment status			
Exercise treatment status	Treatment naïve	Actively treated	Previously treated
Exercise naive	Treatment naive patient	Standard cancer treatment effects	Standard cancer treatment effects
Actively treated with exercise	Exercise as first-line induction or primary monotherapy	Exercise as a first-line or later-line concurrent therapy. Concurrent exercise and cancer treatment may interact to affect treatment response	Exercise as an adjuvant or maintenance monotherapy. Previous cancer treatments may affect exercise treatment response
Previously treated with exercise	Exercise as first-line induction monotherapy followed by surveillance	Exercise as a neoadjuvant or induction monotherapy followed by the primary therapy. Exercise treatment may affect subsequent cancer treatment response	Exercise as part of a previous treatment sequence followed by surveillance

Tumor/disease status and treatment status generate nine distinct clinical oncology scenarios in which exercise could be tested as a new cancer treatment: (a) treatment naïve micrometastases, (b) actively treated micrometastases, (c) previously treated micrometastases, (d) treatment naïve primary tumors, (e) actively treated primary tumors, (f) previously treated primary tumors, (g) treatment naïve metastatic disease, (h) actively treated metastatic disease, and (i) previously treated metastatic disease. In the following sections, we review current observational studies of PA and cancer outcomes from a clinical oncology perspective using the EXACT Framework. We then provide recommendations to improve their clinical utility.

## Observational studies of physical activity and cancer outcomes

A large and growing number of observational studies have examined the associations between PA and cancer outcomes across many different cancer types. These studies have been summarized in numerous systematic reviews and meta-analyses across all cancer types (18, 19) or within specific cancer types such as breast (20, 21), colorectal (22), and lung (23). In the largest systematic review and meta-analysis conducted to date, Friedenreich et al. (18) summarized 136 studies on PA and cancer outcomes including 38 studies on all cancers combined, 9 on multiple cancers, 39 on breast cancer, 19 on colorectal cancer, and 9 on prostate cancer. Most studies included early-stage cancer patients who received heterogeneous treatment protocols (18). Few studies provided any details of cancer treatments beyond whether or not a major cancer treatment modality such as surgery or chemotherapy was received (18).

Self-reported PA assessments generally occurred at variable time points well before or well after the cancer diagnosis (e.g., between 2 to 5 years) and asked patients to recall variable time periods that were unrelated to cancer treatments (e.g., past 6 months, past year, past 10 years). Most studies included a prediagnosis PA measure only (n=88; 65%) while a smaller number of studies included a postdiagnosis PA measure only (n=35; 26%) or both (n=12; 9%) (18). In terms of cancer outcomes, most studies reported cancer-specific mortality only (n=58; 43%) or both cancer-specific and all-cause mortality (n=50; 37%); however, over 20% reported all-cause mortality only (n=28; 21%). Few studies reported on cancer-specific outcomes other than cancer deaths (18). Finally, the most commonly reported subgroup analyses were based on demographic variables such as sex, body mass index, and menopausal status. A few studies reported subgroups based on disease factors (e.g., disease stage, tumor grade, cancer subtype) but almost no studies reported subgroups based on treatments received (18).

Overall, the results showed that higher prediagnosis PA was significantly associated with a lower risk of cancer-specific (HR=0.87; 95% CI=0.82-0.92) and all-cause mortality (HR=0.84; 95% CI=0.80-0.88). Results were also significant for several individual cancer types including breast, colorectal, and hematologic (18). Moreover, higher postdiagnosis PA was even more strongly associated with an overall lower risk of cancer-specific (HR=0.66; 95% CI=0.59-0.73) and all-cause mortality (HR=0.65; 95% CI=0.61-0.71). Postdiagnosis PA was also associated with lower risks for several individual cancers including breast, colorectal, and prostate cancer (18). Subgroup analyses indicated limited effect modification by demographic/health variables with the exception that obesity modified some associations (18).

In the most recent systematic review focused on breast cancer, Cariolou et al. (20) summarized 20 cohort studies that examined the association of postdiagnosis recreational PA with breast cancer outcomes. All 20 studies focused on early-stage breast cancer patients, however, most studies included patients who received very heterogeneous treatment protocols. Moreover, few studies provided any detail about cancer treatments beyond whether or not a major treatment modality was received. A few studies did report the type of surgery (lumpectomy versus mastectomy), however, no studies reported cancer treatment dose or completion (20). PA assessments generally occurred at highly variable time points well after breast cancer diagnosis (e.g., between 2 to 5 years) and referred to variable time periods that were unrelated to cancer treatments (e.g., past 6 months, past year). All studies measured postdiagnosis PA only once (20). In terms of cancer outcomes, most studies reported all-cause mortality (n=17; 85%) and/or cancer-specific mortality (n=12; 60%), however, only 6 studies reported recurrence (20%), and no study reported second primary cancers (20). Finally, the most commonly reported subgroup analyses were based on sex, body mass index, and menopausal status. Few studies reported subgroups based on disease factors and no studies reported subgroups based on treatments received.

Overall, the results showed that higher postdiagnosis recreational PA was statistically significantly associated with a lower risk of all-cause mortality (HR=0.56; 95% CI=0.49-0.64) and breast cancer-specific mortality (HR=0.60; 95% CI=0.47-0.77). No association was found for breast cancer recurrence (20). Subgroup analyses were largely consistent with the overall results; however, one novel subgroup analysis is particularly relevant for the present paper. The authors separately analyzed 4 studies that assessed PA after completion of initial treatment (excluding hormone therapy) based on the rationale that PA after initial treatment may be different than PA during treatment and may be more stable (20). Interestingly, the association of PA performed after initial treatments with breast cancer-specific mortality was nonsignificant and more modest (HR=0.83; 95% CI=0.61-1.12) than the overall association (HR=0.60; 95% CI=0.47-0.77). Although this subgroup analysis has substantial limitations, it does suggest the possibility that PA performed at some point during breast cancer treatment may be more clinically relevant than PA performed well after completion of primary breast cancer treatment.

## Limitations of observational studies from a clinical oncology perspective

In general, systematic reviews and meta-analysis of observational studies of PA and cancer outcomes have concluded that higher prediagnosis PA, and especially postdiagnosis PA, are associated with lower risks of cancer-specific and all-cause mortality overall and for several specific cancer types (18, 20). While these findings have important implications for cancer prevention and survivorship, they provide limited guidance for clinical exercise trials or clinical oncology practice. More specifically, findings relating to prediagnosis PA are particularly uninformative in the clinical context. First, it is very unlikely that any clinical exercise trials will examine the effects of an exercise intervention before a cancer diagnosis on outcomes after a cancer diagnosis. Second, cancer patients are obviously unable to change their prediagnosis PA, therefore, these findings provide limited guidance to cancer patients or clinical oncologists. Nevertheless, findings relating to prediagnosis PA and cancer outcomes may contribute to our understanding of the biological effects of exercise on tumor development and progression.

Unfortunately, even the findings pertaining to postdiagnosis PA have limited clinical utility. Based on the highly variable time periods covered by postdiagnosis PA assessments, it is unclear at which time point postdiagnosis clinical researchers should initiate an exercise intervention. Moreover, recommending “postdiagnosis” PA to cancer patients is not any more clinically useful than recommending “postdiagnosis” chemotherapy or “postdiagnosis” immunotherapy. Cancer treatments are rarely approved to be administered in the “postdiagnosis” setting or at a specific time point postdiagnosis (e.g., between 1 to 2 years). Rather, most new cancer treatments are approved to be administered based on clinical disease events (e.g., newly diagnosed, recurrence, progression) and in relation to existing cancer treatments (e.g., as monotherapy, combined with another treatment, second-line treatment) (24). Based on the EXACT Framework, the following recommendations are made to improve the clinical utility of observational studies on PA and cancer outcomes (**Table 3**).

**Table 3:** Comparison of observational studies of physical activity and cancer outcomes conducted from a cancer prevention/survivorship versus clinical oncology perspective.

Study characteristic	Cancer prevention and survivorship perspective	Clinical oncology perspective (Cancer treatment)
Patient eligibility based on tumor/disease status and treatment status	Patients eligible across tumor/disease status (primary tumor present, primary tumor removed, and even metastatic disease present) and across treatment status (untreated, actively treated, previously treated)	Patients eligible based on a single tumor/disease status (primary tumor present, primary tumor removed, or metastatic disease present) and the same or similar first-line treatment protocol
Cancer treatment data collected	Absent or minimal such as whether or not the major cancer treatment modalities were received (e.g., surgery, chemotherapy, radiation therapy)	Comprehensive including modality, specific type, dose, duration, combination, sequencing, and completion (tolerance) for all cancer treatments received
Number, timing, and focus of physical activity assessments	Single or multiple physical activity assessments conducted at variable or fixed time points before and/or after diagnosis (e.g., 2-5 years), often with wide interquartile ranges (e.g., ± months or years), asking participants to recall variable time periods unrelated to their cancer treatments (e.g., past months or years)	Multiple physical activity assessments conducted at fixed time points in relation to cancer treatments (i.e., before, during, between, and after), with narrow interquartile ranges (e.g., ± days or weeks), asking participants to recall specific cancer treatment-related time periods (e.g., during chemotherapy)
Cancer outcome data collected	Focused on all-cause and cancer-specific mortality, but rarely disease response, recurrence, progression, or other cancer-specific outcomes	All cancer outcomes relevant for a particular clinical oncology scenario such as tumor response (e.g., partial, complete), disease response (e.g., progressive, stable, partial, complete), and multicomponent survival endpoints (e.g., disease-free survival, failure-free survival)
Subgroup analysis performed	Based on demographic/health variables (e.g., age, sex, body mass index, menopausal status) and sometimes disease variables (e.g., disease stage, tumor grade, cancer subtype)	Based on disease variables and cancer treatments including modality, specific type, dose, tolerance, combinations, sequencing, and later-line therapies
Interpretation of associations between physical activity and cancer-specific outcomes	Generic systemic mechanisms with reference to discordant preclinical models (if any)	Specific systemic and mechanical mechanisms based on tumor/disease status (i.e., specific steps in the metastatic cascade) and treatment status (i.e., treatment interactions/synergies) with reference to relevant preclinical models (if available)

## Recommendations for observational studies of PA and cancer outcomes

**Recommendation #1 (recruit clinically homogeneous patients)**: Observational studies of PA and cancer outcomes should recruit cancer patients within a single clinical oncology scenario rather than mixing patients across tumor/disease status and/or treatment status. That is, studies should recruit cancer patients with either the primary tumor removed, the primary tumor present, or metastatic disease present (for a specific cancer) who are scheduled to receive the same or similar first-line treatments. For example, an observational study may recruit newly diagnosed prostate cancer patients scheduled for active surveillance or postsurgical breast cancer patients scheduled for adjuvant chemotherapy. Of course, during follow-up it is likely that tumor/disease status and/or treatment status will change. These key clinical events should be anticipated and incorporated into the study design. For example, some prostate cancer patients on active surveillance will progress and receive treatments such as surgery or radiation therapy. Similarly, some postsurgical breast patients treated with adjuvant chemotherapy will have a distant recurrence and begin treatment for metastatic disease.

Alternatively, a more homogeneous patient population could also be achieved at the analysis stage by restricting the analysis to a particular tumor/disease scenario and/or treatment scenario. Such an analysis would also allow for a direct comparison of the associations of PA and cancer outcomes across tumor/disease status and treatment status by formally testing for an interaction. One limitation of imposing homogeneity at the analysis stage is the risk of having inadequate and/or unbalanced sample sizes in one or more of the disease/treatment scenarios.

**Recommendation #2 (collect detailed cancer treatment data)**: Observational studies of PA and cancer outcomes should collect, analyze, and report detailed data for all cancer treatments beyond simply whether or not the major cancer treatment modalities were received. These key treatment variables should include the modality, specific type, dose, duration (beginning and end dates), tolerance (completion), combinations, and sequencing of treatments (including time between treatments). These data will allow researchers to examine the associations between PA and cancer outcomes in relation to cancer treatments received to determine if and when exercise should be incorporated into an existing treatment protocol. For example, the effects of exercise during or after chemotherapy may be influenced by the specific type, duration, combination, dose, or relative dose intensity of the chemotherapy regimen. Although data are very limited on this issue, the START trial suggested that the effect of exercise during chemotherapy on breast cancer outcomes was numerically better for patients receiving taxane-based chemotherapy and for patients who completed >85% of their relative dose intensity (25). Without more detailed data collection on cancer treatments, these types of analyses and insights would not be possible. Importantly, treatments may change depending on the initial response and are not always planned. Researchers should collect data on all planned and unplanned cancer treatments including second-line and later-line (salvage) therapies.

We acknowledge that collecting such detailed cancer treatment data may pose logistical challenges including limited accessibility/completeness in the electronic medical records, no prior ethical approval, and prohibitive costs. These challenges may be particularly daunting for currently ongoing cohort studies or studies accessing existing medical data bases. Nevertheless, studies with limited cancer treatment data may still improve their clinical utility by applying alternative analytical strategies (see Recommendation #5). Our recommendation to collect detailed treatment data is primarily aimed at new cohort studies of exercise as a cancer treatment that may have access to higher quality electronic medical records containing detailed treatment data. Moreover, the addition of detailed cancer treatment data to cohort studies of PA and cancer outcomes may benefit from new research team members with clinical expertise that have not always been included in such studies (e.g., medical oncologists, radiation oncologists, surgical oncologists, oncology nurses).

**Recommendation #3 (assess PA in relation to cancer treatments)**: Observational studies of PA and cancer outcomes should assess PA in relation to cancer treatments rather than in relation to the cancer diagnosis or arbitrary time periods (i.e., variable time periods before and after diagnosis). The critical cancer treatment-related time periods are before, during, between, and after specific treatments. Therefore, the PA assessments should correspond to the treatment-related time periods (e.g., before chemotherapy, during chemotherapy, after chemotherapy) rather than arbitrary time periods that may traverse multiple treatment-related time periods (e.g., past 6 months, past year, past 5 years). It is important to assess PA for each unique treatment-related time period because the effects of exercise on cancer outcomes and the amount of exercise performed may vary dramatically across treatment-related time periods (26, 27). In some cases, the cancer treatment-related time periods may be too brief to matter biologically and/or to assess PA logistically. One guideline may be to assess PA for any cancer treatment-related time period that lasts at least 4-6 weeks. PA assessments in some clinical settings with limited cancer treatments may be relatively straightforward whereas in other clinical settings with more extensive cancer treatments they may be more complicated.

**Figure 2:**
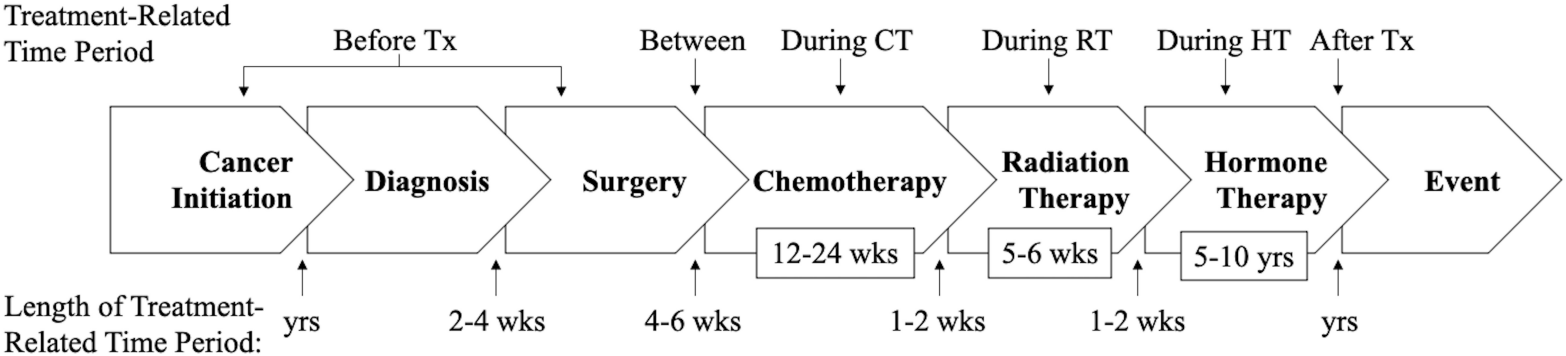
Diagram of the suggested timing of physical activity assessments under a hypothetical cancer treatment protocol. PA, physical activity; Tx, treatment; CT, chemotherapy; RT, radiotherapy; HT, hormone therapy; yrs, years; wks, weeks.

As a simple example, a specific cancer patient group may receive surgery 4-6 weeks after diagnosis, then 12 weeks of chemotherapy 6-8 weeks later, followed by surveillance. In this scenario, an observational study should attempt to collect separate measures of PA between diagnosis and surgery (presurgery), between surgery and chemotherapy (prechemotherapy), during chemotherapy, and after chemotherapy (during surveillance). As a second more complicated example (**Figure 2**), a specific cancer patient group may receive surgery 2-4 weeks postdiagnosis, then 12 weeks of chemotherapy 4-6 weeks later, then 5-6 weeks of radiation therapy 1-2 weeks later, and then 5 years of hormone therapy 1-2 weeks later, followed by surveillance. In this scenario, an observational study should attempt to measure PA between surgery and chemotherapy (prechemotherapy), during chemotherapy, during radiation therapy, during hormone therapy, and after hormone therapy (during surveillance). Ideally, PA assessments should be completed prospectively during each cancer treatment-related time period using self-report or objective measures; however, given logistical challenges it may be more feasible to complete retrospective (recall) assessments after each treatment-related time period has been completed or even after multiple treatment-related time periods have been completed.

Any prediagnosis PA assessment should be devised from a cancer treatment perspective by ensuring that it corresponds to the natural history of the disease rather than lifetime or some arbitrary time point (e.g., past 10 years). Such a measure will be more clinically relevant because it will correspond to the time from cancer initiation until diagnosis. For example, in an observational study of newly diagnosed early-stage cancer patients, cancer initiation may have started several years before diagnosis. A PA measure that captures this time period will be an indicator of whether the tumor/disease has been (unknowingly) treated with exercise. Observational studies should interpret a measure of prediagnosis PA that corresponds to the natural history of the disease as exercise treatment for a treatment naïve primary tumor (if an early-stage cohort) or for treatment naïve metastatic disease (if a *de novo* metastatic disease cohort). At the time of diagnosis, the tumor/disease will either be considered exercise naïve (if patients reported no/limited PA) or previously treated with exercise (if patients reported regular PA). Any previous treatment with exercise may influence the effectiveness of any additional exercise treatment or subsequent biomedical cancer treatments.

In one of the few studies that attempted to measure PA in relation to cancer treatments, Cannioto et al. (28) examined the associations between PA performed before, during, and after chemotherapy with cancer outcomes in 1,340 breast cancer patients participating in a phase III clinical trial. PA was assessed at (a) study enrollment (before chemotherapy) when patients were asked to recall their PA in the month prior to their cancer diagnosis, (b) 6 months after study enrollment when patients were asked to recall their PA during chemotherapy, (c) 1 year after study enrollment when patients were asked to recall their PA for the past year (which would appear to mix during and after chemotherapy), and (d) 2 years after study enrollment when patients were asked to recall their PA for the past year (after chemotherapy). As expected, fewer patients reported regular PA during chemotherapy (28).

In joint-exposure analyses, patients meeting the PA guidelines before diagnosis and at 1-year follow-up (during/after chemotherapy) had a significantly lower risk of disease recurrence compared to patients not meeting PA guidelines at both time points (HR=0.59; 95% CI=0.42-0.82). Similarly, patients meeting the PA guidelines before diagnosis and at 2-year follow-up (after chemotherapy) also had a significantly lower risk of recurrence compared to patients not meeting PA guidelines at both time points (HR=0.45; 95% CI=0.31-0.65). Finally, patients not meeting the PA guidelines before diagnosis but meeting the PA guidelines at 2-year follow-up also had a significantly lower risk of recurrence compared to patients not meeting PA guidelines at both time points (HR=0.54; 95% CI=0.35-0.83). The authors concluded that meeting the minimum PA guidelines both before diagnosis and after treatment appears to be associated with a significantly lower risk of recurrence and mortality among breast cancer patients. These findings suggest that exercise treatment after the completion of chemotherapy (i.e., exercise as a maintenance therapy) lowers the risk of breast cancer recurrence and death regardless of whether patients were treated with exercise before any treatment (i.e., exercise as an induction or neoadjuvant therapy). Such a study provides more clinically relevant information on the role of exercise as a cancer treatment.

**Recommendation #4 (focus on cancer-specific outcomes)**: Observational studies of PA and cancer outcomes should collect, analyze, and report data on cancer-specific outcomes for a given clinical oncology scenario in addition to mortality. These outcomes will be based on the clinical oncology scenario of interest but may include primary tumor response (e.g., partial response, complete response, objective response, major response), disease response (e.g., progression, stable, partial remission, complete remission), recurrence, and endpoints that include primarily or exclusively cancer-specific events (e.g., local or distant recurrence, progression, metastasis, cancer deaths). Although overall survival (death from any cause) is the ultimate outcome, many cancer patients die from cardiovascular disease or other causes that have strong inverse associations with PA (18). If researchers rely exclusively on overall survival, it will be unclear if PA has any benefit as a cancer treatment. Studying PA as a cancer treatment ultimately requires a focus on cancer-specific outcomes (e.g., response, recurrence, progression, death from cancer, death from treatments).

**Recommendation #5 (conduct subgroup analysis based on cancer treatments)**: Observational studies of PA and cancer outcomes should conduct subgroup analyses based on cancer treatments received in addition to demographic/health (e.g., age, sex, body mass index) and disease (e.g., disease stage, tumor grade, cancer subtype) variables. Ideally, these associations should be analyzed for specific cancer treatment protocols if sample size and power permit. If power is limited, observational studies may simply examine the associations between PA and cancer-specific outcomes for PA performed before, during, and/or after individual cancer treatments such as chemotherapy, radiation therapy, or hormone therapy. If power is more adequate, associations between PA and cancer-specific outcomes could be examined in patients who exercised before, during, between, and/or after various combinations of treatments such as chemotherapy plus radiation therapy or chemotherapy plus hormone therapy. Finally, if power permits, associations between PA and cancer-specific outcomes could be examined in patients who exercised before, during, between, and/or after a specific treatment protocol or sequence such as surgery followed by chemotherapy and radiation therapy or neoadjuvant chemotherapy followed by surgery and adjuvant chemotherapy.

**Figure 3:**
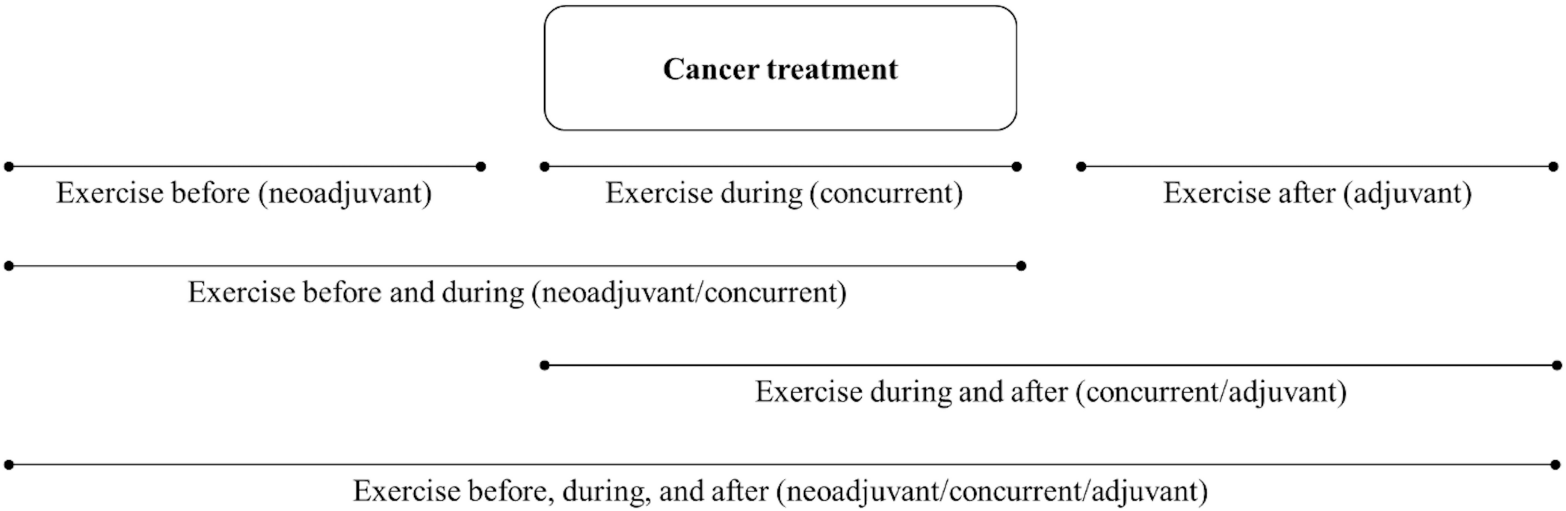
Diagram of possible sequencing of exercise treatment in relation to a single nonsurgical cancer treatment that could be analyzed in observational studies.

Analysis of PA and cancer-specific outcomes should address the entire exercise treatment sequence if possible (i.e., before, during, between, and/or after specific treatments). For example, in the simple scenario of a single nonsurgical treatment such as radiation therapy or chemotherapy, there are 3 distinct treatment-related time periods (before, during, and after) that result in 6 possible exercise treatment sequences (**Figure 3**). Specifically, exercise treatment may occur only before the cancer treatment (neoadjuvant), only during the cancer treatment (concurrent), only after the cancer treatment (adjuvant), before and during the cancer treatment (neoadjuvant/concurrent), during and after the cancer treatment (concurrent/adjuvant), or before, during, and after the cancer treatment (neoadjuvant/concurrent/adjuvant). In the more complex scenario of two nonsurgical sequential cancer treatments, there are 5 distinct treatment-related time periods (before both treatments, during treatment A, between treatments A and B, during treatment B, and after both treatments) that result in 20 possible exercise treatment sequences (**Table 4**). As noted earlier, these types of analyses are most useful to clinical researchers, clinical oncologists, and patients because they provide more precise guidance regarding when exercise should be tested, offered, and/or performed as a cancer treatment in relation to other cancer treatments (i.e., specific combinations and sequencing).

**Table 4:** Potential exercise treatment sequencing options given two (nonsurgical) sequential cancer treatments.

	Before	Treatment A	Break	Treatment B	After
Exercise before treatments	Exercise				
Exercise during A and before B		Exercise			
Exercise between A and B			Exercise		
Exercise after A and during B				Exercise	
Exercise after treatments					Exercise

The above five treatment-related time periods may be combined to produce 20 unique exercise treatment scenarios.

In one of the few studies to perform subgroup analyses based on cancer treatments received, Lee et al. (29) examined the associations between PA performed after surgery only, after surgery plus chemotherapy and/or radiotherapy, and after chemotherapy with or without radiotherapy (and no surgery) with cancer outcomes in 43,596 colorectal cancer survivors from the Korean National Health Insurance Service database. Recent weekly PA was assessed an average of 1.8 years (SD=1.2 years) postdiagnosis making it likely a posttreatment assessment. In stratified analyses by treatment group, PA after treatments was associated with a significantly lower risk of mortality in colon cancer patients who had surgery only (HR=0.75; 95% CI, 0.65 to 0.87) or surgery plus chemotherapy and/or radiotherapy (HR=0.84; 95% CI, 0.73 to 0.97). There was a nonsignificantly lower risk of mortality in patients who did not receive surgery but received chemotherapy with or without radiotherapy (HR=0.74; 95% CI, 0.46 to 1.19), possibly due to a smaller sample size. These results suggest that exercise after various treatment protocols for early stage colorectal cancer may lower the risk of mortality equally across treatment protocols, providing more clinically relevant information about exercise as an adjuvant or maintenance therapy in this setting.

**Recommendation #6 (interpret mechanisms of action based on the clinical scenario)**: Observational studies of PA and cancer outcomes should interpret mechanisms for the associations between PA and cancer-specific outcomes based on the clinical oncology scenario they recapitulate rather than referring to generic mechanisms or discordant preclinical studies. For example, studies that report an association between PA assessed after an adjuvant therapy with a subsequent cancer recurrence in postsurgical patients should discuss how exercise might affect previously treated micrometastases including the survival of DTCs, micrometastasis formation, and metastatic colonization (30). As a second example, studies that report an association between PA and cancer progression in the active surveillance setting should discuss how PA might affect a treatment naïve primary tumor including tumor growth, local invasion, intravasation, survival of CTCs in the blood vessels, and the arresting and extravasation of DTCs (30). Preclinical studies that address the specific clinical oncology scenario should be referenced and discussed, if available.

In simple terms, exercise mechanisms are either biological or mechanical (hemodynamic) and their target is either intratumoral or systemic (**Table 5**). Intratumoral mechanisms are only relevant when tumors have adequate blood supply (i.e., primary or metastatic tumors present). Systemic mechanisms are almost always relevant because it is usually unknown whether a small number of cancer cells may have escaped the primary or metastatic tumors such as CTCs, DTCs, and micrometastases. Biological mechanisms have received the most attention from exercise researchers (31), however, there is growing interest in the role of hemodynamics (31–34). Changes in hemodynamics (especially the location and velocity of blood flow) may have implications for the entire metastatic cascade including tumor growth/invasion, survival of CTCs in the circulation, and survival and growth of DTCs at distant organ sites (35) (**Table 6**). Moreover, these effects may be in isolation (33) or in combination with other cancer treatments (34). In short, exercise may affect how fast blood flows, where it flows, and what is in it (36, 37).

**Table 5:** General categories of mechanisms for explaining how exercise may affect cancer-specific outcomes.

	Biological	Mechanical (Hemodynamics)
**Intratumoral** (only relevant when primary or metastatic tumors with adequate blood supply are present)	Exercise may produce acute or chronic biological effects that enter the tumor microenvironment and independently or in combination with other cancer treatments affect the growth and spread of the tumor	Exercise may produce acute or chronic hemodynamic effects that enter the tumor microenvironment and independently or in combination with other cancer treatments affect the growth and spread of the tumor
**Systemic** (only relevant when small numbers of cancer cells are circulating or disseminated)	Exercise may produce acute or chronic systemic biological effects anywhere in the body that independently or in combination with other cancer treatments affect the survival and growth of small numbers of cancer cells	Exercise may produce acute or chronic systemic hemodynamic effects anywhere in the body that independently or in combination with other cancer treatments affect the survival and growth of small numbers of cancer cells

**Table 6:** Potential hemodynamic effects of exercise based on tumor/disease and treatment status.

Disease/treatment status	Hemodynamics at rest	Hemodynamics during exercise
Untreated Tumor (primary or metastatic)	Many tumors are poorly vascularized leading to poor perfusion and hypoxia	Exercise may increase or decrease blood flow to the tumor which may affect vascularization, perfusion, and oxygenation
Treated Tumor (primary or metastatic)	Tumors with poor perfusion and hypoxia may not respond well to cancer treatments	Exercise may affect vascularization, perfusion, and oxygenation of the tumor which may alter drug delivery and/or response to radiation therapy
Untreated CTCs (from primary or metastatic tumors)	Some CTCs survive circulation, arrest at a distant organ site, extravasate, and become DTCs	Exercise may increase hemodynamic shear stress and affect the number of CTCs that survive circulation, arrest at a distant site, extravasate, and become DTCs
Treated CTCs (from primary or metastatic tumors)	Systemic therapy is effective at reducing the likelihood that CTCs will become DTCs	Exercise may increase hemodynamic shear stress which may interact with systemic therapy to affect the likelihood that CTCs will become DTCs
Untreated DTCs	Most DTCs do not progress to macroscopic metastases even without systemic therapy	Exercise may increase or decrease blood flow to some sites of DTCs which may affect the likelihood that DTCs will progress to macroscopic metastases
Treated DTCs	Systemic therapy is effective at reducing the likelihood that DTCs will progress to macroscopic metastases	Exercise may increase or decrease blood flow to some sites of DTCs which may interact with systemic therapy to affect likelihood that DTCs will progress to macroscopic metastases

## Observational studies of PA within clinical oncology trials as a template

A small number of studies have embedded PA assessments into existing clinical oncology trials (e.g (28, 38–40). This approach has many strengths from a clinical oncology perspective including a narrowly defined patient population based on tumor/disease status and treatment status; detailed collection of cancer treatment data including specific type, duration, and even tolerance; and collection of all relevant cancer-specific outcomes. One advantage of using clinical oncology trials for observational exercise research is that the treatments are usually restricted to a small number of specific protocols (e.g., one standard and one experimental). Such a study addresses a much more clinically relevant question of whether exercise before, during, and/or after specific treatments may improve cancer-specific outcomes. Potential limitations of piggybacking PA assessments on clinical oncology trials are that the assessments may be limited to self-report measures and restricted to specific time points based on the existing trial protocol.

As one example, a large observational study (39) examined the associations between PA assessed during the first month of chemotherapy and survival in 1,218 patients with metastatic colorectal cancer receiving 3 different systemic therapies as part of a phase III trial. Compared with patients engaged in less than 3 metabolic equivalent task (MET) hours of PA per week, patients engaged in 18 or more MET hours per week experienced a 15% lower risk of death (95% CI, 0.71 to 1.02; p_trend_ = .06) and a 17% lower risk of progression (95% CI, 0.70 to 0.99; p_trend_ = .01). This study is an excellent example of using an existing oncology trial to answer a more specific question about exercise as a cancer treatment. Such a finding could potentially inform a phase II or III trial in this setting as well as clinical practice recommendations.

One limitation of this study (39) is that PA was assessed within 1 month of starting chemotherapy and patients were asked to recall their PA over the past 2 months. This recall period includes time before chemotherapy and up to one month during chemotherapy. Consequently, PA was assessed as a partial neoadjuvant/induction therapy and partial concurrent therapy. Moreover, no measure of PA was obtained after chemotherapy completion. Finally, although treatment variables (i.e., planned chemotherapy protocol, prior chemotherapy, prior radiation therapy, and experimental arms) were adjusted for in the analyses, it is unclear whether they were analyzed as subgroups. Future studies of PA in phase III cancer treatment trials may consider PA assessments focused on all cancer treatment-related time periods (i.e., before, during, between, and after) and analyze subgroups based on treatment protocols received.

As another example, PA assessments were incorporated into a 2 x 2 phase III trial comparing an experimental drug versus placebo, and 3 versus 6 months of chemotherapy, in 1,696 stage III postsurgical colon cancer patients (40). Patients were asked to recall their PA over the past two months in the first 3 months of chemotherapy and again at 6 months after chemotherapy. Interestingly, PA was analyzed using cumulative averaging which quantified the time-weighted average of PA across the two time points. The results showed that higher total recreational PA was associated with a lower risk of a disease-free survival event (HR=0.52, 95% CI=0.36 to 0.70). Moreover, in informative subgroup analyses based on treatments received, the authors reported that the association between PA and disease-free survival did not differ based on the experimental arm or length of the chemotherapy.

One strength of this study (40) was the inclusion of PA assessments both during and after chemotherapy. Unfortunately, the analysis of the combined (time-weighted average) time periods did not allow for a separate evaluation of the relative importance of PA during and after chemotherapy. Consequently, the authors were left to conclude that “postdiagnosis” PA was associated with improved survival. Moreover, the study was limited by the recall of the past 2 months rather than the treatment-related time periods of before, during, and after chemotherapy. Specifically, the assessment of PA during chemotherapy included the first 2 months of both the 3 month and 6-month chemotherapy protocols (partially concurrent) instead of the entire chemotherapy periods. Similarly, the assessment of PA after chemotherapy included the past 2 months rather than the entire 6 months since chemotherapy completion (partially adjuvant). Nevertheless, the design and analysis of this study are a major step forward in examining PA from a clinical oncology perspective. Additional studies incorporating PA measures into clinical oncology trials are warranted (9). Moreover, newly developed observational cohort studies should incorporate many of the features of phase III clinical oncology trials into their design.

## Limitations and conclusions

There are important limitations of our review and assessment of observational studies of PA and cancer outcomes. First, our focus was only on exercise as a cancer treatment and not as a supportive care, quality of life, symptom management, disease prevention, or health promotion intervention. Exercise has numerous other health benefits for cancer patients and survivors that affect both clinical research and practice (41). Second, we did not provide any guidance on how to measure PA, how many times to measure PA, or when to measure PA in relation to cancer treatments (e.g., how many times and at what points during chemotherapy or during surveillance). Reviews of PA measurement methods are available elsewhere (42) but the issue of how often and at what time points during treatment-related time periods PA should be measured deserves attention. Third, it is unclear how feasible it will be to measure PA prospectively during every clinically relevant cancer treatment-related time period, especially for complicated treatment scenarios, although wearable technology may provide a solution. In such scenarios, it may be more feasible to have patients recall PA for multiple cancer treatment-related time periods at selected follow-up time points. Fourth, we did not provide any guidance on the statistical analyses of a more complex data set introduced by the numerous exercise treatment combinations and sequences. Fifth, we did not review the specific biological or mechanical (hemodynamic) mechanisms for how exercise might affect cancer-specific outcomes, however, such reviews are available elsewhere (30, 43). Sixth, we relied on existing systematic reviews and meta-analyses rather than conducting our own updated systematic review. Some systematic reviews and notable studies may have been missed; however, we do not believe an updated review would alter the general assessment or recommendations provided in our paper.

In conclusion, most observational studies of PA and cancer outcomes have not been designed from a cancer treatment perspective with the goal of informing clinical exercise trials or clinical oncology practice. Rather, most of these studies have been designed from a cancer prevention/survivorship perspective with the goal of informing public health practice and/or survivorship care. The goal of precision medicine in clinical oncology is to provide the right treatment to the right patient at the right time. The goal of exercise oncology should be the same. Many observational studies of PA and cancer outcomes have contributed to our understanding of the right exercise treatment (e.g., type, dose, intensity) and the right patient (based on demographic and disease characteristics); however, few have contributed to our understanding of the right time beyond “postdiagnosis” (**Table 7**). To achieve this goal, we recommend that future observational studies of PA and cancer outcomes recruit clinically homogeneous patient groups, collect detailed data on cancer treatments, assess PA in relation to cancer treatments, focus on cancer-specific outcomes, conduct subgroup analyses based on cancer treatments, and interpret mechanisms of action based on the clinical oncology scenario that is recapitulated. These simple modifications to the design, analysis, and interpretation of observational studies of PA and cancer outcomes may dramatically improve their clinical utility for researchers, oncologists, and patients.

**Table 7:** Precision medicine in clinical oncology versus observational studies of physical activity and cancer outcomes.

Precision medicine factor	Clinical oncology	Observational studies of PA
Right Treatment (what)	In clinical oncology, the right treatment refers to the specific modality, type, dose, fractionation, and/or scheduling of a cancer treatment (i.e., the treatment prescription)	In observational PA studies, the right treatment (i.e., the exercise prescription) has generally been examined in terms of type (e.g., leisure, occupational, household), dose, and intensity, but rarely in terms of modality (aerobic vs. strength), frequency, duration, progression, or program length
Right Patient (who)	In clinical oncology, the right patient is usually defined in terms of disease factors such as cancer type, subtype, stage, grade, and mutational status	In observational PA studies, the right patient has generally been examined in terms of demographic factors (e.g., age, sex, body mass index, menopausal status) but sometimes has included disease factors (e.g., cancer type, subtype, stage, and grade). In rare cases, the right patient has included mutational status
Right Time (when)	In clinical oncology, the right time is usually defined in relation to disease events (e.g., newly diagnosed, recurrence, progression) and existing cancer treatments (i.e., before, during, or after) including combinations and sequencing	In observational PA studies, the right time has generally been examined in relation to the cancer diagnosis including prediagnosis (e.g., lifetime, past year) and postdiagnosis (e.g., 1 year, 3 years) time points

## Data availability statement

The original contributions presented in the study are included in the article/supplementary material. Further inquiries can be directed to the corresponding author.

## Author contributions

KC conceived the paper and wrote the first draft of the manuscript. CF provided substantive feedback on the literature review, interpretation of the studies, recommendations for future studies, and critically revised the manuscript. All authors contributed to the article and approved the submitted version.
